# Structural Model for Socially Sustainable Public Housing Decision-Making in Chile

**DOI:** 10.3390/ijerph20032543

**Published:** 2023-01-31

**Authors:** Leonardo Sierra, Maximiliano Lizana, Catalina Pino, Amilkar Ilaya-Ayza, Briguitte Neculman

**Affiliations:** 1 Departamento de Ingeniería en Obras Civiles, Universidad de La Frontera, Temuco 4811230, Chile; 2 Efficient Transportation Unit, Ministry of Energy—Government of Chile, Santiago 8340518, Chile; 3 Facultad Nacional de Ingeniería, Universidad Técnica de Oruro, Ciudad Universitaria s/n, Oruro 49, Bolivia

**Keywords:** social housing, social sustainability, SEM, Chile

## Abstract

Normally the social approaches addressed in public housing policies are unclear in the implementation processes. Indeed, public agencies do not have systems that integrate clear social criteria to consider the social assessment of public housing projects. Therefore, the inclusion of social sustainability in planning and early decision-making is limited. In addition, social development technically involves variables that are not normally independent, and its completeness means their relationships must be considered to sum up the impacts. Thus, this work proposes a structural model that explains an integral interrelation of social criteria that determines socially sustainable housing projects for the vulnerable population in Chile. For this, a theoretical model was constructed and validated using a structural equation model (SEM). This system derives from the application of a survey applied to 188 professionals related to the development of public housing. From this, a model of social sustainability of public housing is validated with ten social criteria and eleven unidirectional interrelations, structured in two dimensions: the functional conditions of the home and the environmental conditions of the house. In the first dimension, the relation between the Improvement in family economic availability and Spaces for family development stands out. In the second, the strongest link is between Community health and safety and the Integration of the design in the context.

## 1. Introduction

In recent decades, emphasis in public policies has been placed on the sustainable development of housing; however, the social dimension has been delayed in the early review of projects [1]. This mainly falls to the sustainable formulation of a residential project that must consider the interaction of the spatial and social environment, and the relations that emerge among the actors, the environment and the project. A housing project not only satisfies the basic need for shelter, but also other, more complex needs such as dignity, social and cultural interaction, access to services and quality of life [2]. In Latin America, public programs do not have an integration policy that has successfully reversed the lack of social integration that characterizes vulnerable residential complexes [3]. Examples of this are the border neighborhoods in Tijuana, Nogales and Ciudad Juárez in Mexico [4], the “Working-Class Neighborhoods” in Perú [5] and the “Vertical Ghettos” in Santiago, Chile. These high-rise housing complexes were created to satisfy the demand for social housing, but ultimately, they increased the levels of insecurity and overcrowding of their inhabitants [6]. 

In this vein, the social problem of housing in Latin America lies not only in the lack of infrastructure, but in the qualitative elements that accompany the design and planning of housing with its environment [7,8,9]. To date, theoretical advances have been made on housing-related social criteria; however, these omit integration and interaction among variables and have been limited to the descriptive in the type of assessment [10,11,12,13], which implies a bias in the decision-making.

In the case of Chile, it is common practice for evaluation methods for social housing projects to be centered primarily on technical and socioeconomic criteria [14,15,16]. In these cases, the social aspect focuses on the allotment of housing to future beneficiaries through support and the granting of family housing subsidies. In practice, however, mass housing, the qualitative characteristics for the selection of project alternatives, their location and integration to the context and their coherence with the needs of beneficiary groups do not receive the same treatment [17]. In fact, the national certification of sustainable housing, which includes some aspects of spatial well-being, is recent, and additional funds are needed from the promoter for the development of the process. Objectively, this limits participation in public social housing projects [18].

Similarly, there has historically been a lack of consensus with respect to the social, which constrains the consideration of appropriate social criteria in decision-making [19]. In addition, the social aspects are not naturally isolated, nor do they occur at the same time. First- and second-order social impacts appear in the same context, and even loops that determine states of causality [20,21]. Nevertheless, there are very few documented assessment structures that consider the interaction of factors generated in a social context to estimate the performance of a project. 

Accordingly, the presence of the social aspects and their interactions in the decision-making structure of sustainable social housing projects are currently unclear, as are the methodologies to include the social in early decision-making about design and planning [22,23,24,25]. As a result, the methods for assessing housing projects for vulnerable populations in Chile do not guarantee socially sustainable territorial conditions. Therefore, a conceptual structure is necessary that integrates the social criteria and their interactions and importance to allow decision-making in the planning and design of socially sustainable housing projects for vulnerable populations in Chile.

Consequently, this article proposes a structural model that explains the interrelation of the social criteria that determine socially sustainable housing projects for the vulnerable population in Chile. In the following sections, the state of social sustainability and the social criteria used for the evaluation of social housing projects are introduced. Then the methodology, the research hypothesis, and the latent and observable variables used are presented. The relationships among the variables that represent the social housing assessment model are set out here. Finally, the results are shown and the validated relations and contribution to social sustainability are discussed.

## 2. Literature Review

### 2.1. Social Sustainability in Public Housing

In the beginning, the concept of “sustainability” focused mainly on studies related to climate change and its impacts on the environment. However, when the thought centered on sustainable technology intensified, the debate extended beyond the effects on nature to include urban and social environments. Following these lines, sustainability discourse was linked to that of inequality, since it became more evident that environmental externalities are distributed differentially and disproportionately, both geographically and socially [1]. In this sense, housing projects—and, in particular, for public housing—affect the social sustainability of the built environment. Following Dempsey et al. [26], social sustainability is oriented toward achieving equity by promoting encounters and reducing poverty, and from this the different social strata will benefit from the virtues of economic growth. This implies taking a socially responsible attitude and leaving the following generation a stable world. Thus, social sustainability acquires even greater relevance in public housing meant for the vulnerable population that does not have access to housing through other means. This is the first stage of family and social development in which it is important to protect the environment for a quality of life with dignity [27].

In particular, among the recently established certification processes of sustainable housing in Chile, spatial well-being, social responsibility of the construction process, the conditions of mobility and the cultural relation with the environment are part of the requirements. However, in practice, the certification of these subjects is compromised by the energy and environmental requirements within the total appraisal process of the housing project [18]. 

In other contexts, Fabri et al. [28] make a new proposal of sustainable construction of public housing in Brazil based on the interrelation with the urban space through strategies focused on the urban infrastructure, mobility and access, culture and education, and the generation of income. Their proposals are based mainly on environmentally friendly construction systems with social impact. 

For their part, Karji et al. [29] establish the difficulty in defining the contribution to social sustainability of housing projects given their incommensurability and the peculiarities inherent to the context. After a review of the literature and certification systems, it was determined that the most important social indicators of mass housing projects in Iran are access to basic services (banks, retail, gas stations, hospitals and others), the provision of potable water suitable to the family context, access to mobility and transportation, the quality of the interior of the house and the positive influence on the neighborhood.

Considering the housing policies for the low-income population, Yeganeh and McCoy [30] evaluate the contribution of tax credit programs in the United States. They determine that the cost of living in a house is not equitable in every place, which unequally affects sustainable family and socioeconomic development. The characteristics of the house influence the household economy, and this makes it more or less affordable to live in it. In this sense, they conclude that the cost of the house, energy efficiency, water conservation, health and safety, and the productivity of the built environment are factors that influence the household economy.

On the other hand, Wang et al. [31] anticipate that modern trends in house design are centralized and normally oriented to considerations of esthetic appearance. By contrast, the authors emphasize the need for customer-oriented assessment models that solve the problems of life experiences and interaction with the environment. In this vein, Golic et al. [32] promote the structured participation and communication of the occupants in the renovation of residential buildings. From the analysis of eleven residential projects in Switzerland, nine factors arise that cover the users’ occupational needs. These are early information from the users, user participation in the design process, age-related social and cultural conditions, public and private spaces, spaces for social interaction, relation of density to the urban context, aesthetic, functional and spatial quality, sense of place and improvement in comfort, health and well-being.

Inevitably, infrastructure projects have an impact on the surroundings; however, if they are designed and implemented correctly, they can promote the growth, balance and harmony of society. It is for this reason that in the past few years, social sustainability has been emphasized more in the field of construction and urban infrastructure [10,33]. Particularly, Abdul-Rahman et al. [10] and Golic et al. [32] state that when considering social contributions during the planning and design phases, there are greater opportunities to influence the social performance of the project. For their part, Sodangi [33] notes that in addition to the inclusion of social considerations for the project’s end users, the participatory support and equality of the effects of the project on the community is fundamental.

### 2.2. Social Assessment Criteria for Housing

In exploring the effects of infrastructure on social sustainability, groups of important attributes defined as “assessment criteria” have been categorized for a certain infrastructure type in a geographic and social context [12,34,35,36,37]. The evaluation criteria are configured as latent variables influenced at some stage of the life cycle of the infrastructure by the interest of one or several stakeholders. Normally, these evaluation criteria are measured through observable indicators appropriate to the assessment context and infrastructure type [37,38]. In this sense, Valdes-Vasquez and Klotz [39] categorize five types of social criteria to consider in the planning of construction projects. These are the participation of the interested parties, considerations of the users, formation of teams, management of the considerations and the place of context.

Specifically, Jarafi et al. [12] identify and organize social sustainability criteria for energy modernization projects in buildings. In total, nineteen social sustainability criteria were categorized into six groups: impact on the health and comfort of the occupants; improvement of society; cultural and community education; improvement of the interested parties to the project; improvement of the quality and technology of the buildings; and socioeconomic growth. Likewise, Abed [11] evaluates the social contribution in neighborhood house construction, determining the impact of physical and non-physical factors. The former includes public services, access to opportunities and public space, good quality of services and a connected transport system. The non-physical factors correspond to safety, local social networks, social inclusion, spatial integration, cultural heritage, sense of belonging and identity, community participation and organization. Similarly, Karji et al. [40] classify relevant social indicators in the decision-making of grouped mass residential projects into four criteria: construction and the community; health, safety and risk; habitability; and neighborhood characteristics. 

In Chile recently, some assessment criteria of public housing projects have been studied that contribute to social sustainability for 40% of the most vulnerable population. Barra [41] and Maldonado et al. [8] identify stakeholders associated with public housing projects and the important social criteria for the selection of a sample of residential projects in southern Chile. In this study, ten criteria related to the housing and the neighborhood are included that can be determined based on the information of the planning and assessment processes (See Table 1). Indeed, the social evaluation criteria derived from [8] have been verified in case studies of public housing projects in Chile and the consultation of their beneficiaries. Likewise, interviews with neighborhood leaders were applied in [41] to determine the social criteria for integrating public housing in the cities. In this sense, the criteria presented in Table 1 in this paper consider the needs and aspirations of the community.

In current practice, the consideration of the social criteria in the planning and decision-making of public housing projects is limited to the socioeconomic aspects. In Latin America, it remains for the social aspects to be recognized in housing policies [25]. The recognition of the social implies having observable indicators in housing projects that consider not only the quantitative but also qualitative factors of the implementation context [43]. Additionally, studies that consider the interaction of the social are not common in the decision-making of sustainable housing development planning [32,34]. In this sense, considering a structure of social criteria indicators and their interactions that support decision-making in the matter of public housing implies progress for the development of housing sustainability in Chile.

## 3. Theoretical Model and Hypothesis

### 3.1. Theoretical Model

**Figure 1:**
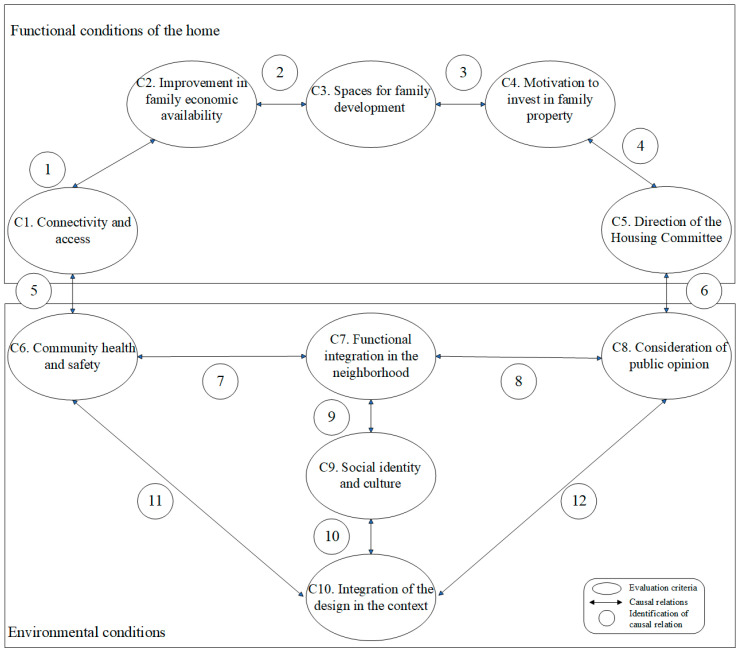
Initial conceptual model among public housing social criteria.

From the previous review section, a conceptual model is proposed that joins together different social housing assessment criteria. First, two dimensions of the assessment criteria are posited that are related and influence each other. These are the *Functional conditions of the home* and *The environmental conditions* (See Figure 1). The social criteria that feed these dimensions have been used in isolated social sustainability evaluations of public housing projects in Chile [8]. In the case of this study, the proposed relations will be validated using a structural equation model. 

Thus, the diagram in Figure 1 illustrates two blocks that relate the environmental conditions to the functional conditions of the home. In a sense, social psychology supports the fact that individual attitudes and homogenous behaviors of family units influence the social thought that affects the environment [44,45]. In another sense, another approach of social psychology establishes that it is the context or environment in which a macrosocial behavior occurs that influences individuals [46]. In this approach, how the thoughts, feelings and behaviors of a family and individual are influenced by the community is analyzed.

The dimension of **Functional conditions of the home** includes social attributes derived from the development of the house and that promote the motivation, progress and family functioning. The criteria that this dimension encompasses are *Improvement in family economic availability; Spaces for family development; Motivation to invest in family property; Connectivity and Access;* and *Direction of the Housing Committee*.

The criterion ***Improvement in family economic availability (C2)*** refers to the facilities of the potential for economic saving that a family can access from initiatives like saving on energy consumption in the home. The criterion ***Motivation to invest in family property (C4)*** refers to the family incentive to make long-term investments in the house [42]. 

***Connectivity and access (C1)*** is a criterion determined by the location of the house and the accessibility of the immediate environment. This criterion refers to the existing basic services in the project that improve the quality of life of families outside the home [24]. These services integrate into the housing project in the urban environment either physically (transport facilities) or by homogenizing the opportunities of the residents (education, work, health, among others). Moreover, the location of the project, connectivity to public transport and facilities for non-motorized modes of transportation (Connectivity and access) can imply a reduction in household spending [47]. 

In the context of public housing in Chile, the creation of social capital is promoted through committees led by neighborhood leaders. These committees manage activities to complete the funding, approval and improvement of the housing project through community participation. In this vein, the criterion ***Direction of the Housing Committee (C5)*** is a component that reflects the proactive behavior and organization of the committee that represents the families who comprise it in a Chilean context [48]. These neighborhood organizations manage the housing project, subsidies, design characteristics and convey the neighbors’ needs to the decision-making stakeholders. The good performance of the Housing Committee can significantly increase the sustainability of the project, contributing social capital through the formation of social networks and reciprocity rules [26,49]. In fact, housing subsidies mean significant discounts in mortgage payments, thermal improvements, extensions or the aesthetic harmonization of neighborhoods, among others. Under this premise, the influence of the *Direction of the Housing Committee* on the *Motivation for family property* is proposed, because the initiatives that the Housing Committee promote make it possible for families to access improvements in their own property at a reasonable cost to their socioeconomic condition [42]. 

***Spaces for family development (C3)*** are associated with the characteristics of the house, with adequate dimensions and conditions of comfort that allow the family to function. The characteristics of the house affect the economy of the family group. For example, a house with thermal insulation has more comfortable spaces and can mean a significant saving for the family economy [50].

The dimension of **environmental conditions** is associated with the physical and abstract characteristics of the neighborhood in which the housing project is located and which permit integration. In this case, the dimension is made up of five criteria: *Community health and safety; Functional integration in the neighborhood; Consideration of public opinion; Social identity and culture; and Implementation of the design in the context.*

Strongly related to *connectivity and access* is the criterion ***Community health and safety (C6)***. This criterion involves aspects of physical safety and health. It considers, and without limiting, the plans of garbage treatment, the capacity of emergency services and health campaigns, among others. The perception of safety contributes to developing a sense of belonging in a neighborhood [26], whereas health is conditional on the place and the practices of good living to create viable communities [24]. 

On the other hand, the ***Consideration of public opinion (C8)*** is a criterion closely related to ***Direction of the Housing Committee***. Indeed, in terms of leadership, having community leaders is fundamental to the degree of participation in stages of the project. A greater degree of participation increases the chances of achieving a harmonic design [51]. 

At the neighborhood level, the project must generate integration for all the needs its residents may have. In this sense, the criterion ***Functional integration in the neighborhood (C7)*** involves design aspects related to universal accessibility. A sustainable neighborhood creates a sense of belonging and long-term identity related to participation in community activities and the generation of local culture [52]. It is also possible to consider from the initial planning and design stages of the project aspects aimed at maintaining or providing a sense of identity and belonging consistent with the context. In fact, local cultures are a resource to confront challenges and find appropriate solutions to community problems and can become a mechanism of social commitment and integration [52]. This is considered in the criterion ***Social identity and culture (C9)***, which is closely linked to the cohesion of the local community by generating points of contact in terms of values, traditions or symbols. 

Another, more concrete aspect is the ***Integration of the design in the context (C10)***. This criterion refers to the immovable elements (multipurpose fields, urban furniture, park lighting, among others) and landscaping that make leisure activities possible and improve the urban image. These elements are indispensable for the coexistence and generation of networks; in addition, they have positive consequences for culture and education [53]. 

### 3.2. Research Hypothesis

Next, the relations between (*R*), the different preconceived latent variables in the conceptual model, are explained (see Figure 1). From Figure 1, validation of the theory is sought in a national context from the correlation of the scores among criteria in agreement with the opinion of specialists.

- ***R1. Connectivity and access/Improvement in family economic availability.***

The suitable location of the housing project can generate an important economic saving in terms of transportation due to its location, connectivity with public transportation and accessibility to non-motorized means [24]. This agrees with the structure of opportunities stated by Kaztman and Filgueira [54] and Frediani [55], where access to certain goods and services provides resources that facilitate access to other opportunities. In addition, it has been seen that the economic availability of families affects the selection of the means of transport and standard of access to services (educational, public–private health) [56].

- ***R2. Spaces for family development/Improvement in family economic availability.***

In Chile, housing subsidies allow vulnerable families to improve the spaces and conditioning of their house [57]. It has been seen that the improvements in the thermal comfort of the house promote the family economy through monetary savings with lower heating fuel consumption [30]. On the other hand, with family economic availability, self-construction for the development and improvement of the spaces in the house is reinforced [58,59].

- ***R3. Spaces for family development/Motivation to invest in family property.***

The improvement in the interior spaces (development of spaces, energy, sound or aesthetic conditioning) is associated with a better quality of family life and, as a result, a concept of long-term investment in the house (family property) [60]. For example, Fahmy [61] specifies that the inner patio is part of the family architectural heritage, being historically a space of meeting in a context of safety and privacy. In [62], the authors establish that home improvements are due to an investment in subcontracted services, domestic production and the time of the owners. According to their study, improvements in American homes occur mainly in homes of older adults that aspire to use them after retirement. Otherwise, this relation can be seen from a commercial approach (sale or rent) in which the pure sense of investment and improvement of the capital gains of the house motivates the improvement of its infrastructure [63].

- ***R4. Direction of the Housing Committee/Motivation to invest in family property.***

In Chile, social housing projects are led by neighborhood groups called Housing Committees. These Committees have leaders who summon the participation of all the beneficiaries and manage collective projects for the improvement of the physical and social living of families through public and/or private financing in houses and neighborhoods. This management begins in the application process, continues in the construction and during the use of the house or neighborhood [17,57]. The functions of the Housing Committees and their community leaders are part of the social inclusion programs established by the Chilean Ministry of Housing. Thus, social capital is established that promotes the collective improvement in the house and the neighborhood. On the other hand, the possession of the house as an asset is reason for participation and belonging to a collective work committee. In some cases, under conditions of transparency, suitable policy rules and legitimacy, the achievements of the Committee result in greater participation and support for the Direction of the Housing Committee [64,65].

- ***R5. Community Health and Safety/Connectivity and access.***

Global characteristics of the project, such as its location, connectivity and access, contribute to the generation of sustainable communities by means of the quality and number of health services available [24]. Indeed, spatial accessibility (time and distance) is commonly taken into account in studies on accessibility to health and safety services. The conditions of access to public transportation, however, also limit access to health services [66]. On the other hand, the conditions of the services also limit accessibility. This is to say, the capacity for medical care, the provision of medical services or the availability and resources of fire and police services determines the degree of accessibility to health and safety from a location point [67,68].

- ***R6. Direction of the Housing Committee/Consideration of public opinion.***

Management by social leaders requires the active participation of the community and that their opinion be considered in the actions they take. This way, the community has the opportunity to assess the Committee Leaders that represents their opinion [69]. Based on suitable leadership and participation, it is possible to strengthen public learning from the consensus of needs to constitute shared beliefs and values from a co-evolution process. This process affects the opinion and behavior that emanates from the members of the community group [70,71]. Otherwise, the result of the management of the community leaders affects their legitimacy with the community through public opinion [69].

- ***R7. Community health and safety/Functional integration in the neighborhood.***

In principle, security is a sociocultural aspect of housing project design [72] that is favored with an inclusive design [73] that provides the accessibility and healthy coexistence within the common areas. Indeed, the dangers associated with the operating conditions of the neighborhood because of design defects or lack of equipment promote accidents, acts of vandalism, improper use of space (uncultivated spaces) and potential impacts on the residents’ physical and mental health [74,75,76]. In addition, in the case of entrenched unsafe conditions, public equipment and spaces cease to provide an efficient service to the community [77].

- ***R8. Consideration of public opinion/Functional integration in the neighborhood.***

Public opinion encompasses different perceptions that could express the needs and perspectives of leisure and integration in a community space. These needs and perspective come together mainly in those public spaces of social convergence [78,79]. In this context, public opinion arises after the experience of the social encounter in the public space. This experience is determined by the functional development of the public infrastructure and equipment under concepts of inclusion, capacity sufficient to demand or a supply of functions adapted to the ages of the users [80]. Otherwise, the planning and design of public spaces and infrastructure also require a suitable citizen consultation to determine the needs of leisure and recreation [81].

- ***R9. Functional integration in the neighborhood / Social identity and culture.***

The standards of sustainable certification promote infrastructure modalities that improve the uses, diversity and capacity to promote an efficient urban design that pays attention to the people and holistic concepts of sustainability [14]. 

Some strategies, such as increasing ground occupation capacity to provide more green areas, sport, recreational infrastructure, and improving the public spaces and/or other equipment, promote cultural integration through built systems, their architecture and functionalities [18]. 

In addition, the actions taken that are conducted in the environment built by the community leave a mark on the place by incorporating the characteristics of the space through cognitive and affective processes, generating a particular symbolic identification to build an urban social identity [82,83,84]. 

Thus, the quality and supply of urban public spaces and equipment give the structural support for the sociability of the residents and the aesthetic qualities favor the formation of a strong neighborhood identity [78,79,84].

- ***R10. Integration of the design in the context/Social identity and culture.***

The urban integration of the projects based on an architectural and functional objective contributes to the formation of the local surroundings, seeking to rescue the existing heritage value and helping to consolidate the local urban image [18]. 

In Chile, the institutional definition of a neighborhood considers the inclusion in a territory where elements of identity and belonging are generated by sharing the equipment, public space and other services [85]. In this vein, Portal [86] anticipates that identity processes are established from the way in which the spaces and their understanding are structured in the development of sociocultural relations.

- ***R11. Community health and safety/Integration of design in the context.***

The design of public residential buildings must consider the social and environmental ecosystem in which it will be inserted. This condition can consider in the project the renovation and revaluation of empty or previously built spaces as a strategy that fosters the safety of the environment. Another form may be the recovery of sites documented as contaminated to drive improvements in public and community health [14]. 

On the other hand, the difficulties in spatial organization and the insertion of a residential complex in cities are reproduced in urban problems of urban disorder, the expropriation of places and crime. The perception of insecurity, fear of crime and violence arises in this view. Indeed, citizen insecurity is manifest in the spatial disorder of the city and its neighborhoods [74]. 

On the other hand, to cope with the increased load of morbidity and health inequalities, the opportunities, risks and challenges for health that adequate urbanization entails must be taken into consideration [87]. Communicable and non-communicable diseases can be prevented by paying attention to the design, construction and management of the environment in which people live. The influence of the built environment is clear in the world evaluation of the morbidity load due to environmental risks that emphasize the need for urban and territorial planning of the integration of new projects enacted in favor of health [87].

- ***R12. Consideration of public opinion/Integration of the design in the context.***

Public opinion is a way to gather and consider the needs of a group of people in the design phases of a project. This contributes to integrating the users of the project, generating a sense of belonging and achieving a design that harmonizes with its users and context [51]. Public opinion is a means to extract information from a geographic context that over the years has developed a process of social co-evolution. This information is substantial to generate housing designs and consistent urban spaces with the beliefs and values of a context [38,88]. In addition, the design located in an urban context that has coexisted with a community constitutes part of its identity. For example, the intervention in spaces that recall family experiences or local historical facts can be valued according to the attitude of the users, their intensity of use and visual sensitivity [89,90].

## 4. Research Method

### 4.1. Structural Equations Models

The structural equation model (SEM) can simultaneously examine a series of relations, making it possible to assess the relation between non-observable constructs, called latent variables. It permits the study of causal relations in non-experimental data. For such models, a sample size over 100 subjects is required, and ideally, over 200 subjects for a greater guarantee [91]. There is no clear rule regarding the sample size for SEM; in general terms, it is promoted that there are at least ten observations for each free parameter. However, sample size can be validated such that SEM significance tests are reasonable. Indeed, sample spaces of restricted size, as is the case of specialists in the area of social housing in a national context, the use of continuous variables, with a normal distribution, with a linear effect, without interaction and with multiple indicators per construct tend to require a smaller number of samples [91].

The structural model of social criteria visualized the correlations and incidences of the observable and latent variables to contribute to the evaluation of social housing in Chile. This helped in understanding the complex relations among the different variables and the factors of influence. The identification of the model was assessed following the two-step identification rules, which involved the individual analysis of measurement and the structural model’s identification. For this, the AMOS 24 software [92] was used to apply SEM during the research process. 

### 4.2. Questionnaire

The theoretical relation among the criteria was validated using a SEM and a database obtained through an online survey applied to professionals with experience in social housing. Specifically, the consultation was directed to the qualified professionals that participate in the decision-making process in social housing planning in Chile associated with the Ministry of Housing and Urban Planning (MINVU) at the central level, and regional agencies derived from MINVU, municipalities, consultants (technical assistance entities), social housing builders and NGOs. In addition, the professionals who participated had to fulfill requirements of education, training and experience. First, a technical or professional education in construction, urban planning or social sciences was required. Second, the focus was on respondents with training in social housing developments. Third, the interviewed respondents had at least 1 year of experience in public housing or associated programs. The survey was applied in 2019 and 2020 and its sample characterization is shown in Table 2. This study is based on a sample of 188 respondents.

The questionnaire was designed according to the evaluation criteria shown in Figure 1 and observable variables (indicators) derived from interviews with experts from public agencies and private social housing project developers. The questionnaire consists of four parts. The first provides the informed consent, requests approval and asks for the respondents’ professional information. The second asks about their experience with public housing developments. The third part is optional, in which the respondent can give contact data to receive the study results. The fourth part asks for the evaluation of the indicators for each public housing criterion in Figure 1. The respondent answers the importance of each indicator for the respective criteria on a Likert scale from 1 to 5, where “1” means No importance/influence and “5” means Extreme importance/influence. 

Once the questionnaire had been designed, it was disseminated nationally to all the technical assistance entities by the Ministry of Housing and Urban Planning (MINVU), a body that ensures the planning and construction of social houses in Chile. For this, the research team disseminated the survey through the internal and external network of MINVU national collaborators. In addition, it was distributed to the professionals in the Social Development Department in the housing area in all the municipalities in Chile, NGOs and universities via e-mail. 

The variables consulted in the questionnaire, as well as their factor loadings, Cronbach’s alpha, Average Variance Extracted (AVE) and Composite Reliability (CR), are shown in Table 3. These parameters are used for the analysis of the reliability of the data and its validity [91]. Generally, it is required that the Cronbach’s alpha coefficient be greater than 0.7 for the model to be accepted. In the case of AVE and CR, the usual thresholds are 0.5 and 0.7, respectively. The factor loading is the weight that relates the indicators to the criterion which they represent by means of a linear regression. The SPSS 24.0 software was used to prove the reliability of the study. 

The characterization of the sample space (Table 2) Is composed of the 188 total answers. Of the ten criteria, three had a Cronbach’s alpha coefficient below 0.7; however, in these cases, when eliminating some of its indicators, the coefficient falls and presents an acceptable factor load, which is why the choice was made to report them [93]. AVE and CR indicated a satisfactory value for most of the criteria, confirming their inclusion in the analysis. With respect to the factor loading of each observable variable (indicator), the lowest value was 0.61 in criterion C7. Thus, each criterion was made up of at least three observable variables (indicators). 

## 5. Results 

**Figure 2:**
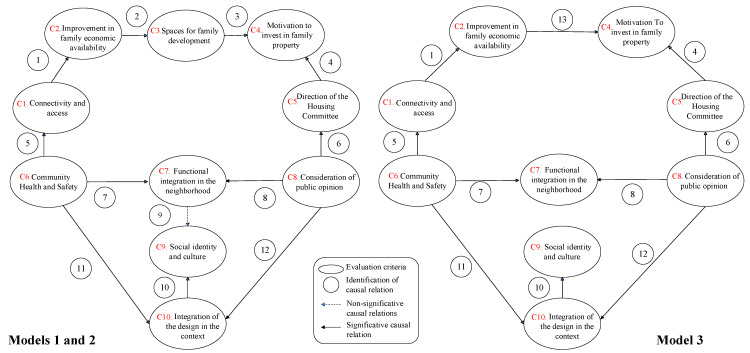
Structures of the evaluated model 1, 2 and 3.

The proposed relations shown in Figure 1 were validated by estimating three structural equation specifications (models 1, 2 and 3). Figure 2 summarizes this graphically, showing the final relationships found. The continuous arrows correspond to significant causal relations in the proposed direction. The only difference between model 1 and model 2 is the second does not include the relationship between *Functional integration in the neighborhood* and Social identity and culture (Relation 9) that model 1 found not statistically significant (represented by the dashed arrow in Figure 2). In addition, model 3 is presented, which is a derivative of model 2 and has a better fit. Model 3 does not consider the criterion *Spaces for family development* (*C3*) in the relationship between criteria *C2* and *C4.* The final model structures were obtained through an iterative process based on the original conceptual framework and the model’s outputs [94]. It must be mentioned that a unidirectional relation between the criteria in Figure 2 is validated in the statistical models. Indeed, the dimension of *environmental conditions* affects the criteria that comprise the *functional conditions of the home*. Global goodness-of-fit measures are presented in Table 4, and statistics of the relationships analyzed are shown in Table 5. 

The model is estimated using maximum likelihood. The reason why we use a method that depends on some distributional requirements is that the literature indicates bootstrap can only be applied when a large sample size is available [91,92], which is not the case in this study. However, as Arbuckle [92] indicates, the multivariate normality of observed variables is a standard distribution assumption in structural equation modeling. The goodness-of-fit statistics in the three models of Figure 2 were similar, indicating that the RMSEA and the Parsimony-Adjusted Measures Index (PNFI) fulfilled the usual criteria (<0.08 and >0.6, respectively). In addition, the values for the Incremental Fit Index (IFI) and the Comparative Fit Index (CFI) were acceptable but still below the typical thresholds (0.9) [95]. This can be explained as a consequence of the limited number of observations available (<200) to carry out the modelling and the associated complexity of the relationship tested (to see a further discussion about the effect of small sample on model fit, please see [96]).

Results of the hypothesis verification test and the fit of the models are presented next. The fit yields significant results among the relations of the latent variables of the model (criteria). In addition, at least ten significant relations are identified with *p*-value less than 0.05. According to Figure 2, model 1 is evaluated with 12 relationships, while in model 2, relationship nine (*R9*) is not considered in the global evaluation. Model 3 provides a better overall fit and considers ten significant relationships without considering the *R2* and *R3* relationships and including the *R13* relationship. In this case, the transitory criterion “Spaces for family development” (*C3*) is not considered. 

From these results, models 2 and 3 emerge as significant alternatives that include variables and relationships that affect the social sustainability of public housing projects in Chile. In this case, model 3 presents a better overall fit according to the estimators in Table 4. However, the high estimator of the relationship *R2* in model 2 cannot be ignored, nor the effect of a limited sample concerning the number of variables [96]. In another line, conceptually, the *Motivation to invest in family property* (*C4*) can be conceived by elements not included in *C3*, such as saving, changing the neighborhood, or acquiring a second home, which are also motivated by the economic availability of the family (*R13*).

From the results, it is observed that the lowest estimator corresponds to that of the causal relation between the variables *Community health and safety* and *Integration of the design in the context* (*R11*). Nevertheless, *Community health and safety* has a greater influence on the criterion *Connectivity and access (R5)* and more still on the criterion *Functional integration in the neighborhood (R7)*. In that sense, there is evidence that community health and safety have a great influence on the environment of homes, thereby having an impact on the integration of the design. 

The highest estimator is the relation between the variables *Consideration of public opinion* and *Direction of the Housing Committee (R6)*. This is associated with the legitimacy of the community regarding the management of its community leaders, which determines the continuity of its operation. Thus, suitable management by the leaders is determined to take into account and channel the committee members’ options. Moreover, the community leaders should promote participation processes to obtain feedback from the community. From this, it may be assumed that once public opinion is considered, the communities trust their representatives more and consolidate their leadership. In addition, this is aligned with *Consideration of public opinion* also significantly affecting the criteria *Functional integration in the neighborhood (R8)* and *Integration of the design in the context (R12).* That is to say, in addition to strengthening *Direction of the Housing Committee*, it facilitates decisions about the environment being implemented under a participatory approach.

On the other hand, it is recorded that the criterion *Direction of the Housing Committee* weakly influences *Motivation to invest in family property (R3)* by promoting access to new complementary subsidies that improve the quality of the house. 

The pre-existing conditions that determine the criterion of *Integration of the design in the context* act on *Social identity and culture (R10)*. This determines that identity and culture are provided mainly through new housing projects harmonically adapted to the broader context that receives them. The inverse effect of the adaptability of the group by itself is still not perceived. That is, the need is perceived for complementary programs related to community infrastructure and housing that allow progressive identity support when the encounter of cultures, between the one arriving and those pre-existing, is very disruptive.

Finally, it is observed that the criterion *Connectivity and access* slightly influences the criterion *Improvement in family economic availability (R1)*. Good connectivity generates a saving in transport for families, supporting economic availability. 

## 6. Discussion

From the results, some theoretical approaches are consistent with the validation of the proposed model in the Chilean national context. First, the firmness of the composition of most of the latent variables stands out. In fact, most proposed relations among the latent variables (criteria) are supported by the results of the structural equation model. Only the relation between *Functional integrity in the neighborhood* and *Social identity and culture* was not sufficiently significant for the Chilean application context. In that sense, it might be possible to envisage that, within the design of the neighborhood surroundings, the characteristics that would influence *Social identity and culture* are linked to harmony and *Integration of the design in the context* and not to the functional characteristics. This contrasts with the strategic proposals of MINVU [18], who promote cultural integration starting with better equipment. Indeed, Valera and Pol [83] and Salazar [84] emphasize the community participation process in the built environment, which generates an identity associated with the design of the infrastructure. However, in the context of the case study, the relation of the community with their neighborhood does not occur in the planning processes, even when the houses are inhabited. Thus, in the context where this study is applied, there are no clear instances that allow this relation.

The relations among the social criteria with the highest estimators are: (R1) Improvement in family economic availability and Spaces for family development; (R2) Consideration of public opinion and Direction of the Housing Committee; and (R3) Integration of the design in the context and Social identity and culture. This is consistent with the national strategies defined in social housing plans where social management entities do a prior monitoring on the families and community leaders in applications for social housing subsidies [17]. This would reflect a balance between the importance of the functional dimension of the home and its environment, reinforcing the hypothesis of considering aspects inherent to the house and community aspects of the families when undertaking a housing project. This could be important background information for the authorities during project evaluations and the impact they can have on communities.

In recognizing these results, it is important to consider the limitations and possible opportunities for improvement from the study. Despite the model having a good fit in general terms, it is advisable to consider the Cronbach’s alpha values of each latent variable, and those observable variables that had low factor loading. Only the composition of the indicators associated with the *C2* (*Improvement in family economic availability*), *C3* (*Spaces for family development*), and *C6* (*Community health and safety*) criteria are not consistent with the recommendation of a Cronbach’s alpha above 0.7 [93]. However, given the proximity of the values, the observable variables are reported since when eliminating some of them the Cronbach’s alpha falls [93]. 

In addition, two observable variables (indicators) with weak factor loading are noted. In the latent variable (criterion) *Functional integration in the neighborhood*, the observable variable (the indicator) *Universal accessibility design* has a value of 0.61; and in the latent variable (criterion) *Motivation to invest in family property,* the observable variable (indicator) *Complementary subsidy stimulates family property* has a factor loading of 0.64. Yet in the first case, the design for universal accessibility affects a minority compared to the other two indicators (*Diversity of equipment and capacity*). Pérez et al. [97] argue the need for “inclusion” within the urban surroundings of society. At the moment, this is a mandate aligned with Chilean public policies [98], and the objectives of sustainable development tend to increase inclusive and sustainable urbanization (ODS11.—Sustainable cities and communities). On the other hand, complementary subsidies are effective public tools with limited access subject to the fulfillment of requirements for families and the availability of public resources. In this sense, it is clear that the subsidy allocation processes are not automatic and require organized work by Housing Committee leaders [42], which has a greater impact and determines the result of granting a subsidy. This is consistent with the approaches by Roque et al. [99], where the importance of social capital over results is recognized. However, in Chile, there are complementary subsidies, which are an incentive to invest in the family property, are often used by housing committees and are assigned [42]. In this light, these indicators justify their participation as part of the proposed model. 

On the other hand, a fair interpretation of the results of this study should consider that there is a possibility that with an increase in the number of observations, more relationships could be uncovered; and those that now were not statistically significant could become significant. However, this does not detract from the relationships validated.

Sustainability is conceived as a holistic model that integrates social, economic and environmental aspects, among others. The problems of fragmented solutions, without considering the multidimensional effects that arise in a habitable ecosystem, are evidenced in “marginal” neighborhoods [100]. In this sense, the results of this work are partial and limited to one dimension of sustainability (the social), which has less development than the other dimensions [14,15,19]. In this case, the contribution is to establish a base that allows an adequate treatment of the social and then relate it to different dimensions in future studies. Likewise, the sense is to advance in future studies towards a holistic and contextualized model following the guidelines of [13,33,38].

The proposed model is appropriate for the processes, culture and operation of public housing projects in Chile. In other contexts, the criteria and indicators suited to the implementation of the current public housing policy must be verified. 

## 7. Conclusions

This study validated a structural equation model based on social criteria applicable to public housing projects in Chile. The model is comprised of ten criteria (latent variables) that address decision-making on social housing and the neighborhood community from the point of view of social sustainability. These criteria are *Community health and safety; Consideration of public opinion; Improvement in family economic availability; Motivation to invest in family property; Connectivity and access; Direction of the Housing Committee; Functional integration in the neighborhood; Integration of the design in the context; Spaces for family development; and Social identity and culture*. In addition, the structure of the criteria is defined by a set of 33 indicators (observable variables). 

In this case, the set of criteria is consistent in affinity with two dimensions: *functional conditions of the home* and, on the other hand, *environmental conditions*. The three strongest relations were distributed in these two dimensions. In the functional conditions of the home, the relations of *Improvement in family economic availability* and *Spaces for family development* stand out. In the environmental conditions, *Community health and safety* and *Integration of the design in the context* exceed their estimator. On the other hand, the link between these two dimensions is attached to the relation between *Consideration of public opinion* and *Motivation to invest in family property*.

This study is a support for decision-making by public agencies charged with the allocation of social housing. Through these criteria and the interrelation structure, it is possible to advance in the determination of the social contribution among housing projects. This contributes to the sustainable assessment and planning of public housing from the social point of view. 

Future lines of enquiry could extend the model to the application of public housing case studies as well as integrating a socioeconomic and environmental model. 

## Figures and Tables

**Table 1:** Social criteria determined in the planning of public housing in Chile (Source: Adapted from [8,41,42].

Social Criterion	Description
Family economic availability	Aspects that imply a change in the family income. For example, elimination of rent, transportation expenses, energy saving, among others.
Motivation to invest in family property	Incentive for families to have their own house and to invest in it.
Space for family development	Suitable dimensions of the space of a house, such that the family members can undertake their activities appropriately.
Direction of the Housing Committee	This refers to the management of the committee directors (community leaders) that promotes action to access public housing subsidies. It is focused on the union of the organized group that constitutes social capital.
Connectivity and access	Access to the different types of basic services that enable an optimal quality of life (includes access to schools, health centers, safety, public transport, leisure infrastructure, among others).
Community health and safety	Every aspect that contributes to health and safety in the neighborhood. For example, garbage treatment plans, capacity of emergency services close to the neighborhood, among others.
Functional integration in the neighborhood	The capacity and diversity of urban infrastructure to enable entertainment, cultural–social development and the inclusion of physically challenged people in the neighborhood.
Consideration of public opinion	This refers to all the opinions of the families in the application process, planning and selection of the type of house. Projects prepared participatively are considered.
Social identity and culture	This refers to promoting cultural relevance in the neighborhood according to the social and cultural diversity of the families, and the presence and valuation of the historical or natural heritage.
Integration of the design in the context	This refers to the harmonic design of a project, so that it does not disturb the landscape or is adapted to the wider context in which it is located.

**Table 2:** Characterization of the respondents.

Type	Characteristic	Quantity	Percentage
Academic level	Technical	7	4%
	University	144	76%
	Graduate studies	37	20%
Professional	Construction engineer	58	31%
	Social worker	37	20%
	Architect	42	22%
	Others	50	27%
Work Experience	1 to 2 years	26	14%
	3 to 10 years	86	46%
	11 to 15 years	36	19%
	More than 15 years	39	21%
Institution	(Consultant) Technical assistance entity	76	40%
	SERVIU ^1^	39	21%
	MINVU ^2^	3	2%
	Builder	13	7%
	Municipal	10	5%
	Others	47	25%

Note: (1) Housing and Urban Planning Service (Executive agency of MINVU); (2) Ministry of Housing and Urban Planning.

**Table 3:** Reliability of the indicators for the study.

Criterion	Indicator	Factor Loading	Cronbach	AVE	CR
C1	Frequency of public transport.	0.66	0.746	0.57	0.84
	Distance to public and retail services.	0.81			
	Capacity of adjacent services (health clinic, schools, police).	0.82			
	Accessibility for ecological modes of transport (walking, cycling, etc.).	0.72			
C2	Saving through good connectivity and transport offering.	0.68	0.601	0.56	0.79
	Saving in heating.	0.79			
	Saving by leasing or dividend.	0.77			
C3	Distribution of a more customized space.	0.71	0.630	0.52	0.76
	Heating.	0.71			
	Outside noise.	0.74			
C4	Existence and influence of an organizing committee.	0.87	0.747	0.68	0.86
	Committee promotes the enhancement of family property.	0.89			
	Complementary subsidy stimulates family property.	0.64			
C5	Number of activities by the committee.	0.86	0.753	0.66	0.85
	Tenure of community leaders.	0.88			
	Percentage of support to the community leaders.	0.69			
C6	Community space equipment (lighting, benches, etc.).	0.68	0.646	0.49	0.79
	Absence of uncultivated (not equipped) spaces in the environment.	0.70			
	Access to emergency services (health centers, police, etc.).	0.72			
	Geographic security of the location.	0.69			
C7	Diversity of equipment (benches, sports equipment, etc.).	0.87	0.710	0.63	0.84
	Capacity of equipment.	0.88			
	Universal accessibility design.	0.61			
C8	The selection of attributes of the committee’s family diagnosis record.	0.80	0.770	0.68	0.86
	Free opinion of the committee members.	0.87			
	The percentage of committee agreement.	0.80			
C9	Diversity and culture.	0.89	0.800	0.72	0.88
	Contextual historical and cultural heritage.	0.82			
	Diversity and empathy.	0.83			
C10	Participatory design.	0.75	0.727	0.57	0.84
	Harmony of design by policy conditions.	0.74			
	Design harmony through environmental disturbance study.	0.81			
	Design harmony through architecture of the project.	0.72			

**Table 4:** Models’ goodness-of-fit.

Goodness-of-Fit Statistics	Model 1	Model 2	Model 3
RMSEA	0.066	0.066	0.065
PNFI	0.613	0.614	0.639
IFI	0.818	0.819	0.846
CFI	0.814	0.815	0.843
Degrees of freedom	483	484	394
Chi-squared	879.750	879.888	700.270

**Table 5:** Estimation of the models.

Relation	Variables			Model 1		Model 2		Model 3	
				Estimate (t-Statistic)	*p*	Estimate (t-Statistic)	*p*	Estimate (t-Statistic)	*p*
R1	Connectivity and access (C1)	/	Improvement family economic availability (C2)	0.475 (4.66)	***	0.476 (4.66)	***	0.346 (3.73)	***
R2	Improvement family economic availability (C2)	/	Spaces for family development (C3)	0.951 (4.77)	***	0.951 (4.77)	***	-	-
R3	Spaces for family development (C3)	/	Motivation to invest in family property (C4)	0.435 (3.04)	0.002	0.435 (3.04)	0.002	-	-
R4	Direction of the Housing Committee (C5)	/	Motivation to invest in family property (C4)	0.264 (4.33)	***	0.264 (4.33)	***	0.366 (5.56)	***
R5	Community health and safety (C6)	/	Connectivity and access (C1)	0.531 (4.07)	***	0.531 (4.07)	***	0.443 (3.62)	***
R6	Consideration of public opinion (C8)	/	Direction of the Housing Committee (C5)	0.937 (6.27)	***	0.939 (6.28)	***	0.969 (6.45)	***
R7	Community health and safety (C6)	/	Functional integration in the neighborhood (C7)	0.690 (5.14)	***	0.692 (5.15)	***	0.671 (5.06)	***
R8	Consideration of public opinion (C8)	/	Integration of the design in the context (C10)	0.420 (4.55)	***	0.420 (4.55)	***	0.442 (4.72)	***
R9	Functional integration in the neighborhood (C7)	/	Social identity and culture (C9)	0.033 (0.40)	0.686	-	-	-	-
R10	Integration of the design in the context (C10)	/	Social identity and culture (C9)	0.875 (5.45)	***	0.909 (5.92)	***	0.875 (5.48)	***
R11	Community health and safety (C6)	/	Integration of the design in the context (C10)	0.258 (2.97)	0.003	0.265 (3.13)	0.002	0.235 (2.79)	0.005
R12	Consideration of public opinion (C8)	/	Integration of the design in the context (C10)	0.685 (5.58)	***	0.684 (5.58)	***	0.701 (5.63)	***
R13	Improvement family economic availability (C2)	/	Motivation to invest in family property (C4)	-	-	-	-	0.305 (3.88)	***

Notes: Consider ‘***’ as values less than 0.001; and ‘-’ as relationships not considered for models 1, 2 or 3.

## Data Availability

Data are available under request to the corresponding author.
